# Increased water availability at various timescales has different effects on stomatal closure point in isohydric piñon pine and anisohydric juniper

**DOI:** 10.1038/s41598-025-00582-6

**Published:** 2025-05-12

**Authors:** Vachel Kraklow, L. Turin Dickman, Max G. Ryan, Emma Lathrop, Jack Heneghan, Dea Musa, Sanna Sevanto

**Affiliations:** 1 Earth and Environmental Sciences Division, Los Alamos National Laboratory, Los Alamos, NM 87545 USA; 2 Integral Ecology Group, Los Alamos, NM 87545 USA

**Keywords:** *Pinus edulis*, *Juniperus monosperma*, Isohydric, Anisohydric, Drought, Water availability, Plant ecology, Plant physiology, Stomata, Ecophysiology

## Abstract

Stomatal Closure Point (SCP) has commonly been used to describe drought response strategies in plants, with isohydric species maintaining relatively high, constant SCP compared to anisohydric species that can lower SCP with increasing drought severity. However, there is evidence that, within these groups, SCP may respond dynamically to environmental conditions. Here, we explored how increasing water availability affects SCP in classically isohydric piñon pine and anisohydric one-seed or Utah juniper at various spatial- (i.e., from branch, to tree, to ecosystem) and temporal- (i.e., hours to decades) scales. Our results show that short-term increases in water availability decreased SCP in isohydric piñon pine, making it more anisohydric, while short-term rehydration had no effect on SCP in anisohydric juniper. Increasing mean annual precipitation, on the other hand, increased SCP in both species. Our findings are consistent with documented differences in the use of ABA to control stomata in iso- and aniso-hydric species on short timescales, and with structural acclimation in both species at long timescales. These results illustrate that the local environment plays a large role in determining SCP.

**Supplementary Information:**

The online version contains supplementary material available at 10.1038/s41598-025-00582-6.

## Introduction

Stomatal Closure Point (SCP), or the leaf water potential (Ψ_leaf_) at which stomatal conductance (g_s_) approaches zero, is the point at which a plant stops photosynthesis to preserve water during drought. Based on how plants regulate Ψ_leaf_, SCP has commonly been used to classify species drought response strategies (i.e., desiccation avoidant or isohydric, and desiccation tolerant or anisohydric^1^). While the mechanisms underlying these different strategies are debated, current theory suggests an important role of plant hydraulics^2,3^. Specifically, species with a desiccation avoidant drought response strategy (i.e., isohydric behaviour), are often associated with higher xylem vulnerability to embolism^4^. Leaf water potential in these plants declines to a relatively high (i.e., less negative; typically − 1.5 to − 2.5 MPa^2,5,6^), constant mid-day leaf water potential (Ψ_md_) associated with afternoon stomatal closure in avoidance of excessive embolism and loss of hydraulic function under drought^2,7,8^. When drought severity increases such that pre-dawn leaf water potential (Ψ_pd_) declines to Ψ_md_, species with isohydric behavior no longer open their stomata in order to preserve water. On the other hand, species with a desiccation tolerant strategy (i.e., anisohydric behavior), are often associated with lower xylem vulnerability to embolism, and can keep stomata open under more severe drought^9^. In anisohydric species, Ψ_md_ declines with increasing drought so that the slope between Ψ_pd_ and Ψ_md_ is ≥ 1 until stomata no longer open (g_s_ = 0 and Ψ_pd_ = Ψ_md_), which typically occurs at relatively low (− 3 to − 6 MPa) minimum Ψ_md_ (Ψ_min,_ SCP)^2,10,11^. However, recent debate on how plants are classified as isohydric or anisohydric^2,5^ and how SCP is defined suggests that, beyond intrinsic plant hydraulic traits, the local environment might play a larger role in determining SCP than previously thought^3^.

The physiological or biochemical mechanisms that actually trigger stomatal closure during drought are linked to leaf turgor loss point (Ψ_tlp_)^9,12–14^, but these mechanisms might differ between plants with different SCP. In conifers, for example, Pinaceae and Araucariaceae that tend to close stomata at relatively higher water potentials rely on the plant hormone abscisic acid (ABA) to induce stomatal closure. Cupressaceae, on the other hand, typically close stomata at more negative water potentials, using high water tensions to drive stomatal closure^12,13^. These differences in stomatal response, along with observed differences in the plasticity of turgor maintenance between isohydric piñon pine and anisohydric juniper^15^, suggest that SCP could be affected by tissue hydration or, as with Ψ_tlp_, sample rehydration protocol during measurements at short timescales^15^.

Interestingly, there is evidence that the current approach used in iso- vs. aniso- hydric classification may not consider SCP response to increases in water availability. For example, previous studies have shown a decline in Ψ_md_ in isohydric species under waterlogged conditions^16,17^. Additionally, in the experiments performed by Sevanto et al.^18^, the authors found that piñon pine minimum leaf water potential (Ψ_min_) taken as Ψ_md_, often considered a proxy for SCP^2,19^, declined with consistent watering in the control treatment of the experiment (unpublished data). This is of particular interest as piñon pine is classically categorized as an isohydric species^20^, and therefore expected to maintain relatively constant Ψ_min_ as water availability changes. Together, these results suggest that environmental factors can shift SCP more rapidly than previously thought, and that isohydric plants can shift to more anisohydric behavior in response to increased water availability.

Based on the findings of Sevanto et al. (unpublished), we set out to test how increasing water availability affects SCP in classically isohydric piñon pine (*Pinus edulis* Engelm.) and anisohydric one-seed or Utah juniper (*Juniperus monosperma* Engelm., or *Juniperus. osteosperma* Torr.) at different temporal and spatial scales of rehydration. These species have become icons of plant hydraulic studies because of their coexistence across large areas in the arid and semi-arid climate zones of the southwestern U.S. and their different stomatal closure points^10,11,15,18,21–24^. Based on current understanding of stomatal responses and their triggers, we hypothesized that rehydration on short timescales (minutes, to days, to weeks) would decrease SCP in piñon pine, consistent with declining ABA^12,13^, and increase SCP in juniper, consistent with increasing Ψ_tlp_^15^. On long timescales (decades), we hypothesized that SCP in both species would increase (become less negative) with increasing water availability due to structural acclimation^25^. We compared the tree-level results of shifting Ψ_min_ from Sevanto et al. (unpublished) to our own measurements of SCP determined from benchtop dehydration of field collected branch samples provided with varying levels of supplemental hydration, as well as SCP calculated from literature-based meta-analysis of stomatal conductance and water potential from sites across the Southwest U.S. with varying mean annual precipitation. This allowed us to explore how SCP is influenced by spatial- (i.e., from the branch, to tree, to ecosystem) and temporal- (i.e., minutes, to hours, to seasons, to decades) scales of increased water availability.

## Results

### Piñon SCP declines with increased branch hydration

**Fig. 1:**
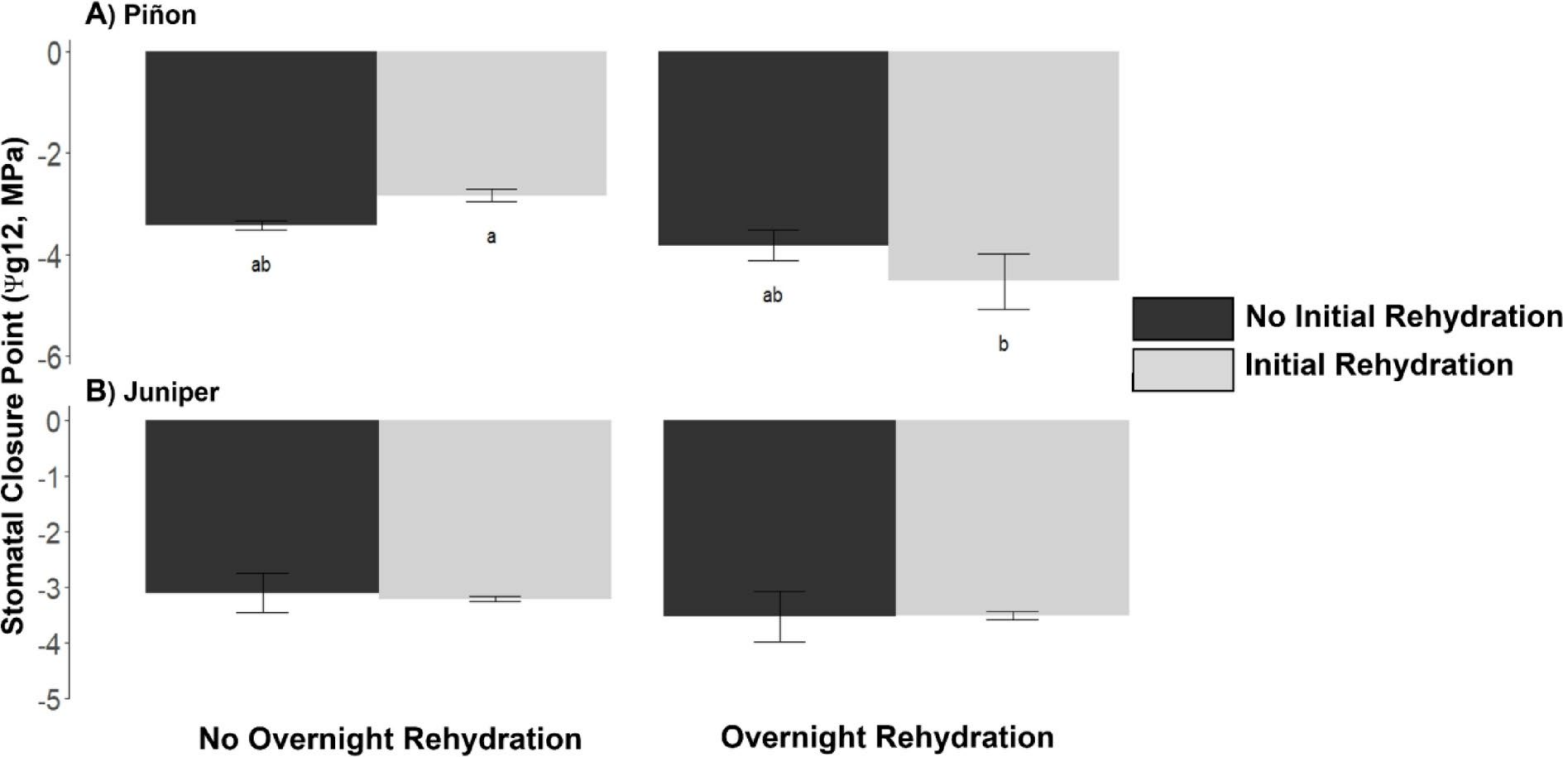
Piñon SCP declines with increased branch hydration. Piñon SCP significantly decreased by more than 1.5 MPa (*p* = 0.03) with “Overnight Rehydration” relative to “No Overnight Rehydration” in the “Initial Rehydration” treatment (**A**). Juniper SCP did not change with rehydration (**B**). Error bars are standard errors. Lower case letters indicate significant differences at *p* = 0.05.

Our branch-level rehydration experiment showed that overnight (hours), or initial rehydration (minutes), alone did not change piñon pine SCP. However, when combined, overnight and initial rehydration together significantly decreased piñon pine SCP by 1.5 MPa (*p* = 0.03) relative to initial hydration alone (Fig. 1a). Juniper SCP did not change with overnight rehydration relative to no overnight rehydration, or with initial rehydration relative to no initial rehydration, with or without overnight rehydration (Fig. 1b).

### Piñon Ψ_min_ declines after months of regular watering in pots

**Fig. 2:**
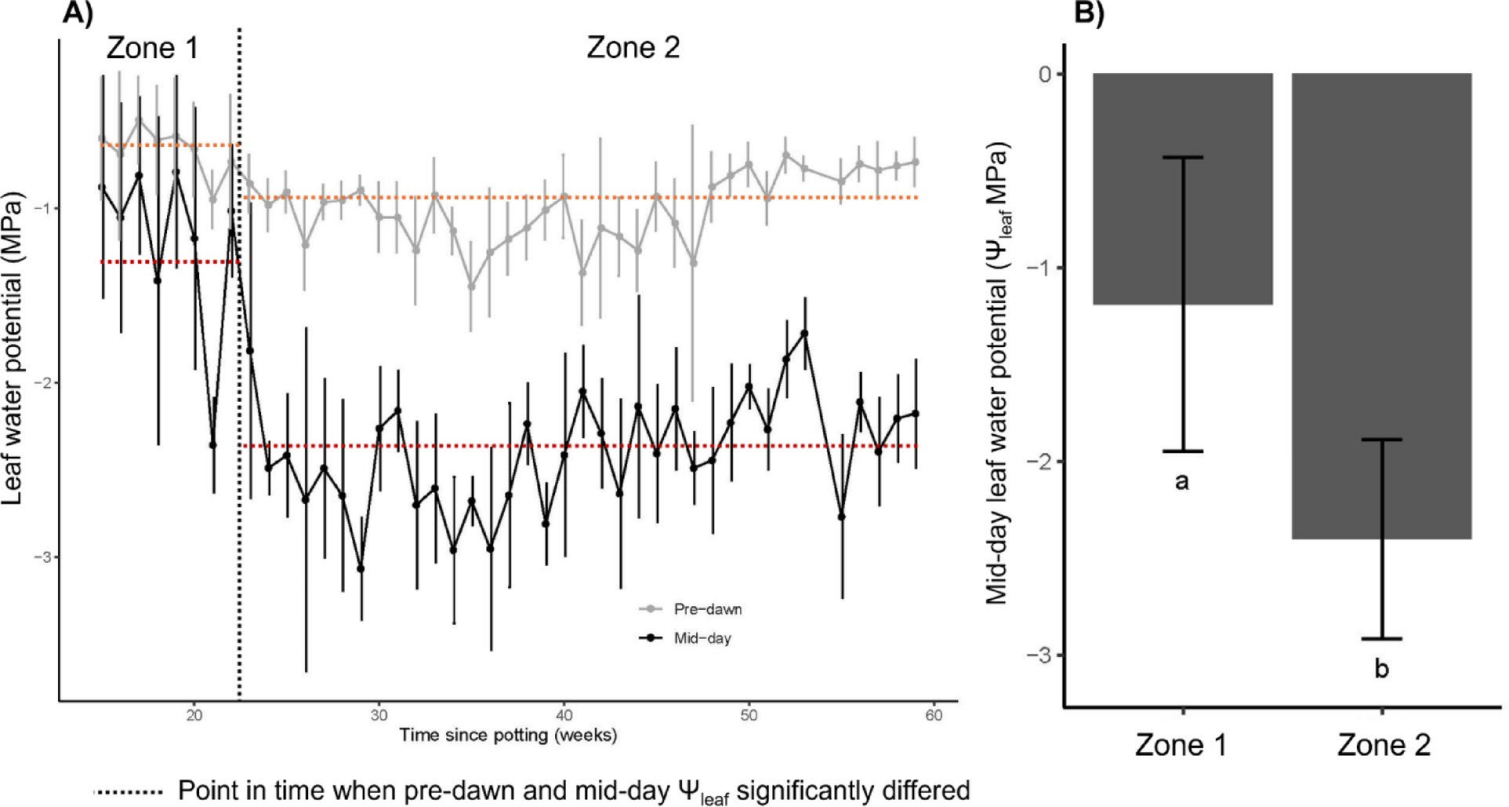
Piñon Ψ_min_ declines after months of regular watering. Pre-dawn (Ψ_pd_) and mid-day (Ψ_md_, Ψ_min_) leaf water potentials of five potted mature piñon pine trees irrigated daily for over one year and measured weekly after acclimatizing for a three month (14 week) period (**A**). After 5 months of regular watering (“Zone 1”), piñon Ψ_min_ declined by over 1.2 MPa (*p* < 2.2e^−16^), and this decrease was maintained for the duration of the study (8 months; “Zone 2”; **B**). Vertical black dashed line in panel (**A**) indicates the point after which pre-dawn (orange dashed line) and mid-day (red dashed line) Ψ_leaf_ differed significantly for more than two consecutive weeks and Ψ_md_ remained significantly lower than Ψ_pd_. Error bars are standard deviation. Lower case letters indicate significant differences at *p* = 0.05.

Analysis of the tree-level rehydration data (control treatment from Sevanto et al.^18^) showed that ~ 22 weeks into the experiment, average piñon pine Ψ_min_ declined by 1.21 MPa, from − 1.19 (Zone 1) to − 2.40 MPa (Zone 2; *p* < 0.0001) (Fig. 2). This decrease in piñon pine Ψ_min_ was maintained for the duration of the 8 month study, suggesting a shift in SCP. The decline in Ψ_min_ resulted in a significant increase in ΔΨ, the difference between Ψ_pd_ and Ψ_md_, from 0.63 ± 0.68 to 1.41 ± 0.46 (*p* < 0.001) from Zone 1 to Zone 2.

### Piñon and juniper SCP increase with water availability across the landscape

**Fig. 3:**
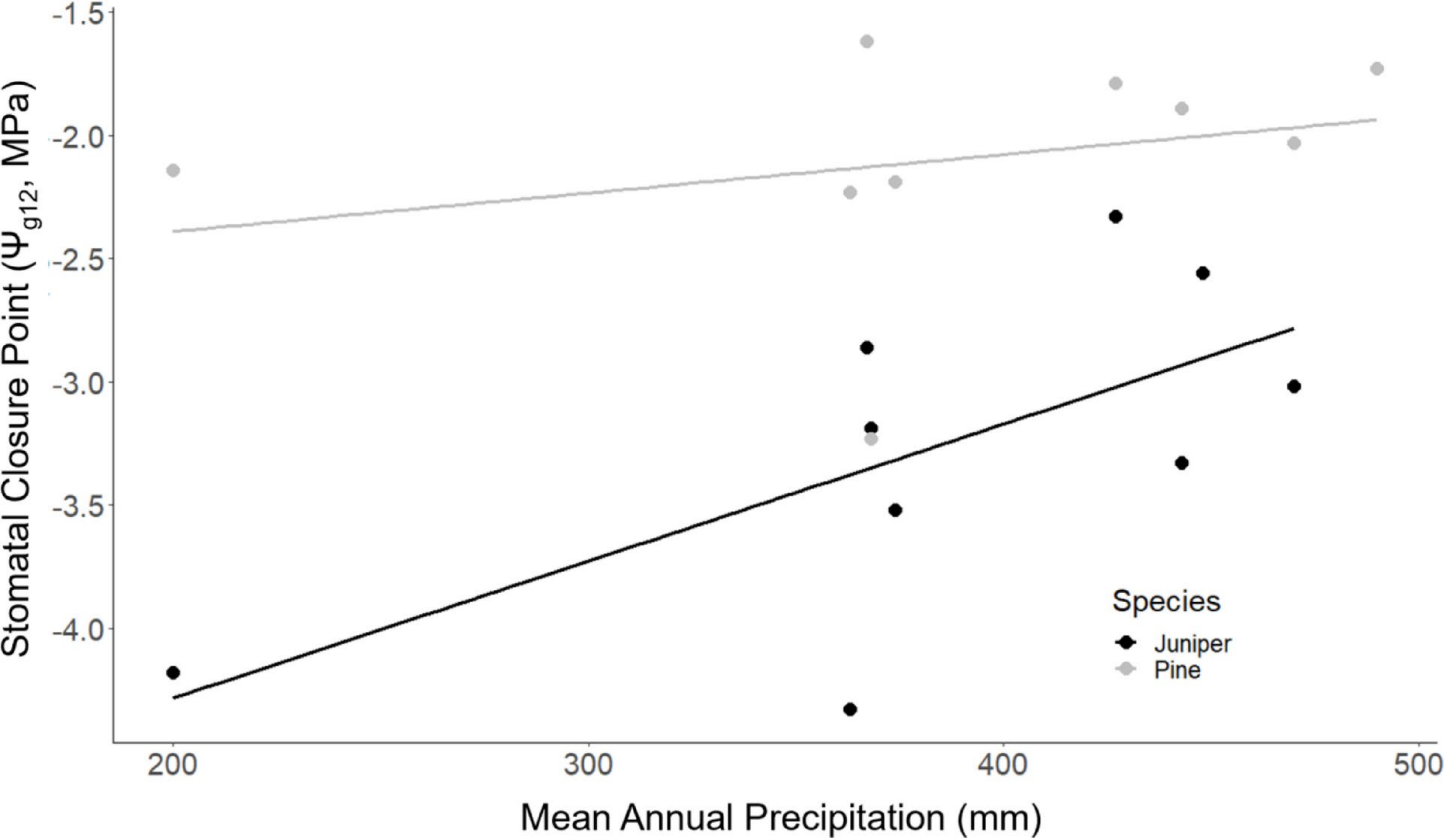
Piñon and juniper SCP increased with water availability across the landscape. Juniper (black) and piñon (gray) SCP both increased with increasing mean annual precipitation (MAP). Slopes did not differ significantly between species (*p* = 0.2, Table S1) and SCP increased, on average, by 0.35 MPa with a 100 mm increase in MAP (*p* = 0.05, Table 1).

Analysis of SCP responses to water availability at the ecosystem-scale showed that SCP increased with increasing mean annual precipitation (MAP) for both species (Fig. 3). Growing season precipitation (July–September; *p* = 0.13; SI Table 1) showed no correlation with SCP in either species. The rate of change in SCP with MAP did not differ significantly between species (*p* = 0.2, SI Table 2), increasing by 0.34 MPa with a 100 mm increase in MAP across both species (*p* = 0.05; Table 1). This relationship was robust even when the two lowest MAP values (~ 200 mm) were excluded from the analysis. SCP still increased by 0.6 MPa with 100 mm increase in MAP (*p* = 0.077). The full model including both MAP and species explained 59% of the variation in SCP (*p* = 0.0005; Table 1). Both MAP (partial η^2^ = 0.112) and species (partial η^2^ = 0.512) had large effect sizes, with Species having a larger effect on SCP, consistent with the significant differences in y-intercepts between species (*p* = 0.0003) and their iso-/aniso-hydric classifications (Table 1).

**Table 1:** Summary of linear model coefficients for SCP as a function of mean annual precipitation.

	Estimate	Std. Error	pr( >|t|)	numDF
Intercept	− 4.578361	0	2.94e−06	15
Mean Ann. Precip	0.003435	0	0.046546	15
SpeciesPine	1.147303	0	0.000332	15

lm(P_g12_ ~), adjusted R^2^ = 0.5923, *p* = 0.0004675.

## Discussion

**Fig. 4:**
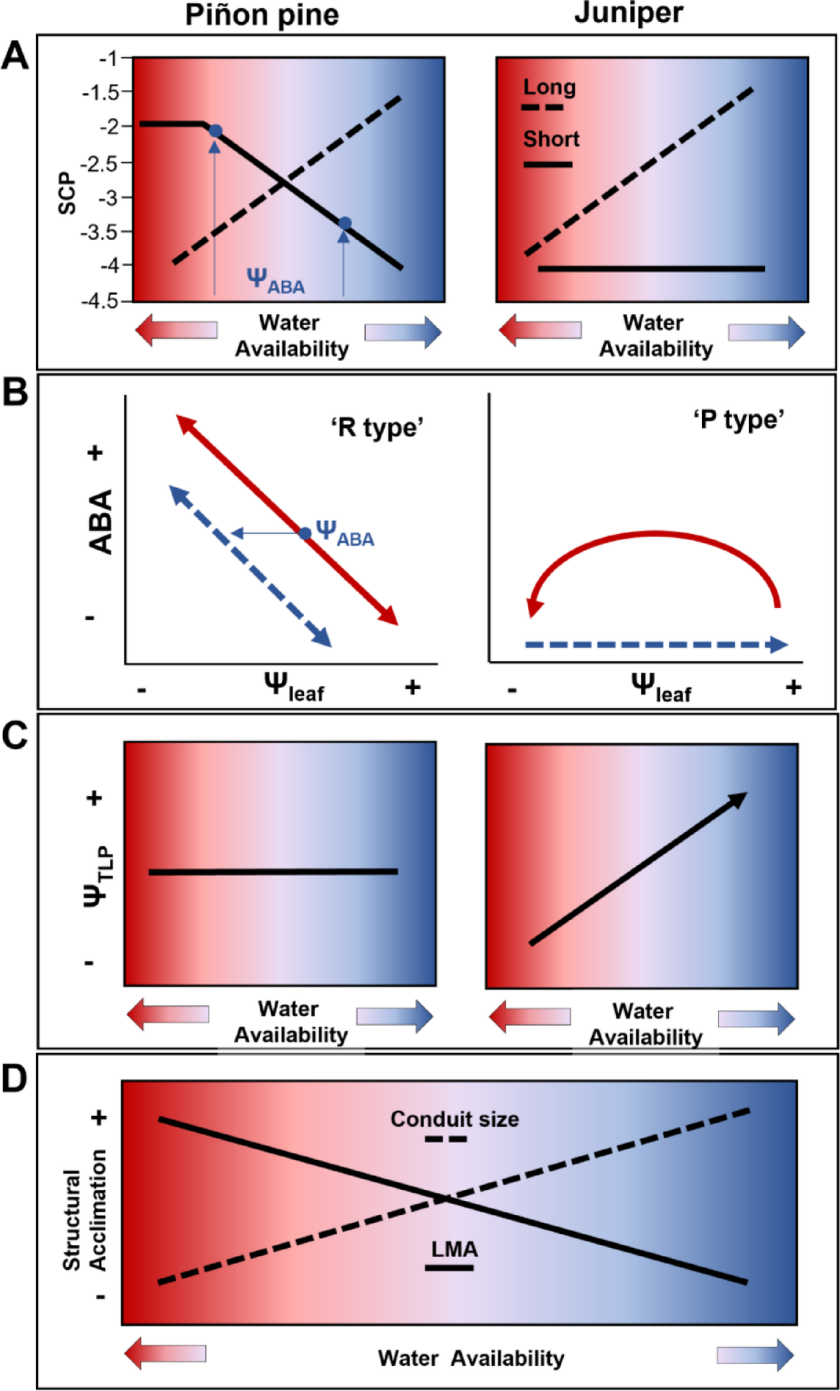
Hypothetical variation in SCP with water availability at various timescales. (**A**) On short timescales in piñon pine (solid line), increased water availability decreases SCP, possibly by shifting Ψ_ABA_ (blue arrows), the trigger point for ABA production. (**B**) On short timescales (solid red line) under decreased leaf water potentials (Ψ_leaf_), ABA increases in ‘R type’ species, like piñon pine (left panel), while ABA briefly increases, peaks and then declines in ‘P type’ species like juniper (right panel). Under rehydration (dashed blue lines), a more negative Ψ_leaf_ is required to trigger the same amount of ABA production in ‘R type’ species while ABA remains at low values in ‘P type’ species. (**C**) Piñon pine is unable to adjust turgor loss point (Ψ_TLP_) (left panel), however, Ψ_TLP_ increases with increasing water availability in juniper (right panel). (**D**) In both piñon pine and juniper, structural acclimation may occur on both short and long timescales, increasing SCP in both species (**A**, dashed line).

Our results show that SCP is influenced by increased water availability, but the magnitude and direction (more or less negative) depend on the plant species and the spatial- (i.e., branch to ecosystem) and temporal- (i.e., minutes to decades) scales across which the water was available (Fig. 4a). Specifically, consistent with our hypotheses, our results show that increased water availability decreased SCP in piñon pine at the branch and tree-level in response to rehydration at the scale of hours to weeks, but increased SCP at the ecosystem- or decadal-scale. For juniper, however, SCP was influenced by increased water availability only at the ecosystem- or decadal-scale, contrary to our first hypothesis. It is generally thought that SCP in isohydric species, such as piñon pine, is constant with limited influence of the growth environment (but see Sade et al.^26^ and Zhao et al.^27^ for shifts of isohydric species to anisohydric behavior under drought), while in anisohydric species SCP can decline with increasing drought^2^. Our results, however, show that SCP of isohydric piñon pine can decline by over 1 MPa with short-term (hourly to seasonal) rehydration, making it behave in a more anisohydric fashion, while increase in short-term water availability had no effect on anisohydric juniper SCP (Fig. 1).

The decline in piñon SCP with increased water availability at shorter timescales suggests that SCP in this species can respond more rapidly to favorable environmental conditions than previously assumed. This finding may be linked to the greater hydraulic plasticity that has been demonstrated for isohydric species^28^. This plasticity could involve adjustments in hydraulic conductance, facilitated by the expression and regulation of cell membrane aquaporins, which has been suggested as the mechanism enabling SCP decline under drought in anisohydric species^15^. Under conditions of ample water availability, upregulation of aquaporins enhances hydraulic efficiency, supporting increased water flow and contributing to lower SCP. To eliminate the effects of growth environment and tree geno- and pheno-type on SCP responses, we used side branchlets of a single larger branch to test effects of rehydration on SCP. Replication of this experiment with more branches from iso- and anisohydric species are needed to make the findings conclusive. However, our branch and tree-level findings are consistent with the observed declines in Ψ_md_ with waterlogging in other isohydric species such as wild cherry (classified as isohydric^16,29^), or Gamhong apple (the genus *Malus* have been described as isohydric^30–32^). The magnitude of the declines in Ψ_md_ in these studies (~ 2–2.5 MPa) is consistent with our observations in piñon pine. While these studies did not directly link their findings to implications for SCP, combined with our results, they suggest that elevated water availability at short timescales can result in increased anisohydricity of species typically classified as isohydric.

This has implications for metrics used to classify (an)isohydricity at short timescales. For instance, a larger ∆Ψ_leaf_ between midday and predawn indicating weak stomatal regulation^2,33^ is often used to separate anisohydric from isohydric behavior under drought conditions. Yet, based on our observed increase in ∆Ψ in Zone 2 (Fig. 2), pinon pine would be classified as anisohydric. Therefore, patterns in water availability should be considered when using ∆Ψ to characterize a species as isohydric or anisohydric. Interestingly, based on the g_s_ measurements reported in Sevanto et al.^18^, the piñon pines in the tree-level rehydration experiment did not close their stomata at midday in either Zone 1 or Zone 2 (Fig. 2). This suggests a physiological shift in how piñon pine regulates Ψ_md_ independent of stomatal closure, which could be related to changes in turgor and ABA regulation through membrane permeability (i.e., aquaporins)^28^. This further complicates the use of Ψ_md_ as an indicator of SCP in absence of g_s_ data. However, the decline in Ψ_md_ after consistent watering supports the findings of Sevanto et al.^34^ that show a decrease in piñon pine SCP when inoculated with fungi that increase water transport capacity.

While our study did not investigate the mechanisms responsible for the differences in plasticity of SCP between piñon pine and juniper, existing knowledge from previous studies can be used to provide mechanistic explanations for our observations, and develop new, testable hypotheses. Specifically, the difference in responses of piñon pine and juniper to short timescale rehydration could be related to new observations of the differences between isohydric and anisohydric species in use of ABA to induce stomatal closure^12,35,36^ and/or with differences in turgor maintenance under drought^15^ (Fig. 4b,c).

Based on how ABA controls stomatal aperture during closure and reopening, seed plants can be divided into two groups: rising, or ‘R type’ (i.e., exhibiting entirely ABA-mediated stomatal control^12,13^), and peaking, or ‘P type’ (i.e., ABA levels increase to initiate stomatal closure, but stomatal closure maintenance eventually becomes hydraulically-mediated^37^). ‘R type’ stomatal control has been associated with isohydric and ‘P type’ with anisohydric desiccation control/tolerance (Fig. 4b). Generally, ABA biosynthesis is triggered by a loss of cell turgor in the leaves as they dry out during periods of high-water stress, with peak ABA biosynthesis occurring when Ψ_leaf_ is close to leaf Ψ_tlp_^38^. The ability to alter Ψ_tlp_ as a result of short-term (a few hours) rehydration has been observed in juniper but not in piñon pine (Fig. 4c)^15^ suggesting more plasticity in SCP in juniper than in piñon pine, contrasting with our results.

However, to change the Ψ_leaf_ at which stomata close (i.e., SCP), the plant can either maintain constant Ψ_tlp_ but alter the Ψ_leaf_ that triggers ABA biosynthesis (Ψ_ABA_), shifting the turgor loss safety margin (i.e., distance between Ψ_ABA_ and Ψ_tlp_), or it can maintain the turgor loss safety margin but change Ψ_tlp_, or both. Piñon pine’s inability to adjust Ψ_tlp_ at short timescales, along with ‘R type’ ABA control, could result in a shift in Ψ_ABA_ with increasing water availability (increasing turgor) (Fig. 4b) towards the structurally defined Ψ_tlp_, leading to a decrease in SCP with increasing water availability (Fig. 4a). With declining water availability (declining turgor), the inability to adjust Ψ_tlp_ would lead to constant short-term SCP (isohydric behavior). In juniper, on the other hand, the observed lack of plasticity in SCP in response to short-term rehydration could be related to its ability to use osmotic adjustment to alter Ψ_tlp_ at timescales similar to our bench dehydration (a few hours)^15^. In this case, it can be hypothesized that the plant has more plasticity to quickly adapt in order to maintain function under decreasing water availability (anisohydric behavior), but increasing water availability does not directly impact SCP because Ψ_tlp_, and consequently SCP, decreases in synchrony with the dehydration process (Fig. 4a,c) until the structurally determined SCP is reached (Fig. 4a). A more negative Ψ_tlp_ is typically associated with a more “anisohydric” strategy^39^. Therefore, it is suggested that by lowering the Ψ_tlp_, anisohydric species, like juniper, are able to keep their stomata open, thereby maintaining photosynthetic activity at more negative Ψ_leaf_^40^.

Alternatively, the short-term decline in SCP in the tree-level measurements could be driven by other mechanisms such as the root system regulating stomata through hydraulic signals (see Carminati & Javaux^41^). Interestingly, our tree-level results on piñon pine agree with the branch-level results suggesting that root signals alone were not responsible for the decline in piñon SCP, or that they did not override leaf-level signals if opposing them. Future research investigating the greater SCP plasticity in piñon pine than juniper in response to changing water availability at diurnal to seasonal timescales and its possible connection with differences between ‘R type’ and ‘P type’ ABA response and Ψ_tlp_ plasticity is needed to test these hypotheses.

At longer timescales, our results support an increase in SCP with increasing water availability across both species consistent with structural acclimation to the growth environment^42^ (Fig. 4d). Typically, SCP correlates with xylem vulnerability to embolism^4,43^, with more cavitation-resistant anisohydric species adapted to more water-limited environments^12^. But species on both ends of the (an)isohydry continuum can acclimate to maximize water use and productivity by increasing xylem conduit diameter (lower flow resistance) and decreasing leaf mass per area (LMA) in less water-limited environments^44–48^. This could lead to increased xylem vulnerability to embolism and consequent increase in SCP^49^. Additionally, previous studies have shown that prolonged exposure to higher environmental humidity (i.e., water availability) can reduce leaf hydraulic capacity. This reduction is primarily attributed to the development of less effective water-conducting tissues through effects on the vascular pathway^50^. Such structural adjustments could, in turn, lead to an increase in SCP over longer timescales. Whether through shifts in LMA, conduit size, or other structural changes such as reduced leaf hydraulic capacity, our results support the hypothesis of structural changes as the driver for adjustment of both piñon pine and juniper SCP with water availability on long timescales^51^ (Fig. 4a,d).

Because SCP controls the drought severity at which the plant closes stomata and moves from a carbon sink to a carbon source under drought, plasticity at different timescales has implications for a wide range of ecosystem responses to environmental changes. Our results suggest that increased long-term drought could decrease SCP in both piñon pine and juniper via structural increases in drought tolerance. This could help maintain tree vitality beyond current model predictions^52^ that do not account for such acclimation. A decrease in SCP as a response to drought could also maintain the total annual ecosystem carbon balance under the changing environment even if carbon allocation between above- and belowground systems might change.

These long-term benefits, however, will be superimposed on short-term changes and their impacts on plant function in these arid ecosystems where plant growth is heavily influenced by precipitation during the North American Monsoon (NAM). While the intensity of monsoonal moisture from NAM has increased over the last few decades^53^, future predictions of end of the century monsoonal moisture over the southwestern U.S. vary^54–57^. If monsoonal moisture availability continues to intensify (i.e., extreme, short-term precipitation events), our results suggest that the plasticity of SCP could benefit piñon pine, allowing utilization of additional short-term moisture to keep stomata more open and grow faster. This opportunistic behavior, however, could lead to consequences for structural hydraulic vulnerability that could be detrimental with intensifying drought and heat waves^58,59^. Therefore, a deeper understanding of the mechanisms and timescales of both SCP plasticity and changes in water availability are needed to predict the future of southwest U.S. piñon-juniper woodlands and similar ecosystems around the world.

## Conclusion

We found that increased water availability at short timescales caused a decline in piñon SCP, but it did not affect SCP in juniper. At long timescales, increased mean annual precipitation was associated with higher SCP in both species, consistent with structural acclimation. Our findings at short timescales may be attributed to differences in the use of hormonal signals (i.e., ABA) to control stomata in iso- and anisohydric species on timescales of hours to days. Relative plasticity in leaf turgor loss point and the leaf water potential which triggers ABA production, combined with the R- and P-type ABA use in isohydric and anisohydric plants, may explain our observed differences in plasticity of piñon and juniper SCP at short timescales. On longer timescales, our results suggest that increased drought could decrease SCP in both piñon pine and juniper via structural increases in drought tolerance. These results illustrate that the local environment plays a large role in determining SCP. However, depending on moisture availability from the North American Monsoon, the plasticity of SCP could benefit piñon pine if monsoon moisture continues to intensify. Intensifying drought and heat waves, however, could impact structural hydraulic vulnerability. Understanding the mechanisms and timescales of both SCP plasticity and changes in water availability are needed to better understand the future of southwest U.S. piñon-juniper woodlands.

## Methods

To test our hypotheses, we evaluated variation in SCP in response to increased water availability in three independent studies across a range of scales from branch to tree and ecosystem.

In the branch-level experiment, SCP was determined by generating stomatal dehydration response curves for branchlets collected from excised branches of mature, field-grown piñon and juniper (*Juniperus monosperma* only) measured both immediately after sampling and after short-term rehydration (i.e., hourly to daily). To eliminate the effects of growth environment and tree geno- and pheno-type on SCP responses, one large, south-facing, mid-canopy branch (~ 1–1.5 cm diameter, ~ 50 cm length, ~ 3 m height) was excised underwater (tap water) using cutting shears from a healthy, mature piñon pine. Branches were collected at the Mesita del Buey study site (34.30° N, 106.27° W; elevation 2140 m a.s.l) piñon–juniper woodland in northern New Mexico, within the Los Alamos Environmental Research Park^60^ at pre-dawn on 28 and 29 October 2019, respectively. They were collected from approximately 70–80 year old mature, dominant trees (see e.g. Rich et al.^61^). The area has a temperate montane climate, with mean annual temperature 9.2 °C (25 year mean; 1987–2011), January being the coldest month (− 2 °C on average) and July the warmest month (20 °C). Mean annual precipitation (1987–2012) is 415 mm of which roughly 50% falls during the North American Monsoon season from July to September (Los Alamos Weather Machine http://environweb.lanl.gov/weathermachine/). The collected branches were placed in a bucket of tap water and transported to the laboratory (approximately 10 min away), where six branchlets (~ 3–4 mm diameter) were harvested from each branch using shears for immediate measurement (herein “No Overnight Rehydration” treatment). The large branch was then re-cut under water and kept in a bucket of tap water in a greenhouse under natural light overnight (herein “Overnight Branch Rehydration” treatment) for measurement the following morning when another six branchlets were harvested for measurement. For SCP measurement on branchlets in both the Overnight Rehydration and No Overnight Rehydration groups, three of the six branchlets were fitted with Teflon tubes filled with tap water and left to sit for approximately 15 min to increase hydration levels for the initial stomatal conductance and water potential measurement (herein referred to as “Initial Rehydration”), and three were measured with no additional treatment (herein referred to as “No Initial Rehydration”). This allowed us to determine whether SCP can shift in response to rehydration at hourly to daily timescales.

SCPs were measured using the standard benchtop dehydration method that examines the relationship between g_s_ and Ψ_leaf_ to generate stomatal dehydration response curves^62,63^. Following excision and rehydration tube installation (for Initial Rehydration treatment only), we measured g_s_ using an infrared gas analyzer (Li-Cor 6400, Licor Inc. Lincoln NE, USA). Measurements were conducted with the following settings using the 2 X 3 LED chamber: PPFD 1500 µmol m^−2^ s^−1^, CO_2_ 400 ppm, T_leaf_ 20 °C, and relative humidity of 10% to match the laboratory conditions. Immediately after the g_s_ measurement, we removed the initial rehydration tube (when applicable) and measured Ψ_leaf_ using a Scholander-type pressure chamber (Model 1005, PMS Instrument Company, Albany, OR, USA). These measurements were repeated consecutively as the sample dried until g_s_ = 0. We then fitted a Weibull function to the g_s_ response curve:

1 $$g_{s} \left( {\% \text{ of }g_{max} } \right) = 100 - \frac{100}{1 + {e^{a\left( { \Psi_{ leaf} - {\Psi_{g50} }}\right) } }}.$$

where g_max_ is maximum g_s_, $a$ is a fitted parameter describing the slope of the curve, Ψ_leaf_ represents measured leaf water potential, and Ψ_g50_ is Ψ_leaf_ at 50% loss of g_max_. We then extracted the fitted parameter ($a$) to calculate Ψ_g12_, or Ψ_leaf_ at ∼12% of maximum g_s_, as a proxy for SCP.

In the tree-level experiment, we used unpublished midday (minimum) leaf water potential (Ψ_min_)^19^ as a proxy for SCP of mature, piñon pine trees transplanted into pots in a greenhouse and exposed to regular watering (see control treatment in Sevanto et al.^18^). Specifically, five mature, 2–2.5 m tall piñon pine trees were transplanted in 86-L pots and irrigated daily to field capacity with tap water. During transplantation, the original soil around the roots was preserved as well as possible to maintain good soil-root contact and avoid root damage. The empty space in the pots around the original soil was filled with potting soil. The plants grew under natural light with the greenhouse temperature controlled between 15 °C (nighttime) and 35 °C (daytime). The plants were not fertilized during the experiment. After acclimatizing for a three-month period, Ψ_leaf_ was measured on two branches per tree using pressure chamber (Model 1005, PMS Instrument Company, Albany, OR, USA)^64^, at pre-dawn (Ψ_pd_) and mid-day (Ψ_md_) weekly for approximately ten months, from 25 March 2010 to 2 Feb 2011^18^.

**Fig. 5:**
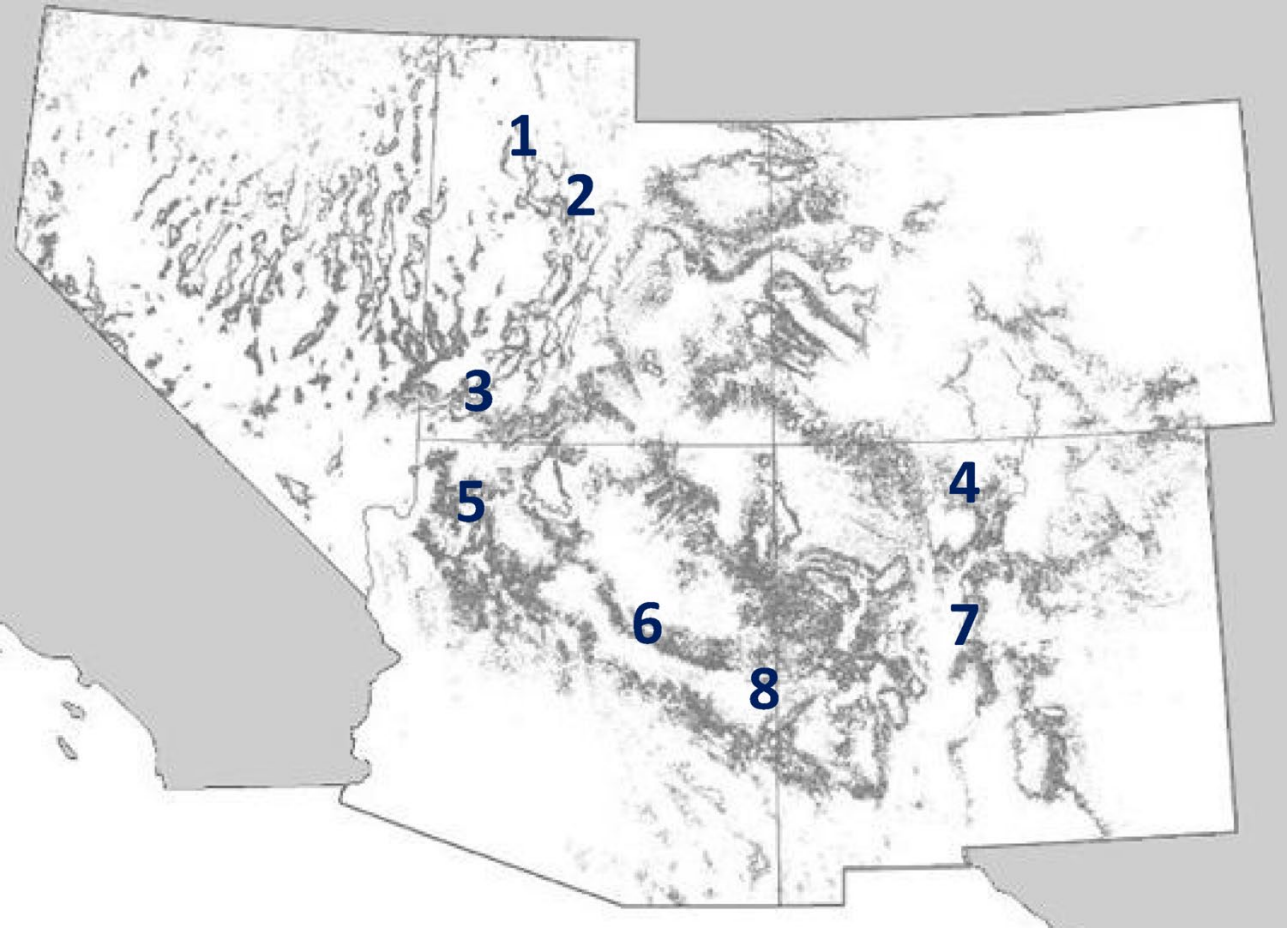
Site locations used in the ecosystem-scale analysis. 1) Tooele, UT; 2) Birdseye, UT; 3) Zion, UT; 4) Pajarito Plateau, NM; 5) Grand Canyon, AZ; 6) Pinedale, AZ; 7) Los Pinos Mountains, NM; 8) Blue, AZ. See Table 2 for more site information.

In the ecosystem-scale study, we generated stomatal dehydration response curves from published g_s_ and Ψ_leaf_ data for naturally occurring piñon pine and juniper trees at 8 sites across the species range varying in elevation and annual precipitation (see Table 2, Fig. 5). Data from Limousin et al.^22^ included drought and irrigation manipulations, further expanding our precipitation range. Data were extracted using WebPlotDigitizer (https://automeris.io/WebPlotDigitizer/). To determine SCP, Eq. 1 was fitted with a Weibull function to extracted data by site, species and treatment, where applicable, and SCP was approximated from the fitted parameters as in the branch-level experiment. We used Ψ_pd_ as the leaf water potential metric instead of Ψ_md_ here because Ψ_pd_ was available from more sites over a larger climate range than Ψ_md_. Because of the use of Ψ_pd_, our estimates for SCP from these studies are conservative in that the drought inducing closure reflected in Ψ_pd_ is severe enough to prevent stomatal opening completely rather than inducing only an afternoon closure reflected in Ψ_md_. To test for effects of water availability on SCP, we then evaluated relationships between cross-site SCP and mean annual (MAP) and growing season (July–September) precipitation reported in the publications. MAP was calculated differently within each of the three publications. Williams and Ehleringer^21^ reported long-term averages from stations near the study sites calculated from NOAA climatological data annual summaries for Arizona and Utah. Limousin et al.^22^ reported MAP averaged over a 30-year period between 1991 and 2011, while Garcia-Forner et al.^11^ used MAP averaged over a 25-year period between 1987 and 2012. As each study used a long-term average which spans decades, evaluating changes in SCP across the landscape and based on decadal mean precipitation and precipitation manipulation treatments implemented over the course of a decade allowed us to determine whether SCP can shift as a response to changes in water availability at decadal timescales.

**Table 2:** Description of sites used in the ecosystem-scale analysis.

Site	Species	Lat. (°N)	Long. (°W)	Elevation (m)	MAP (mm)	Jul-Sep Precip. (mm)	References
1. Tooele, UT	J	40.5	− 112.3**	1980	448	79	^1^
2. Birdseye, UT	PJ	39.9	− 111.5**	1860	368	80	^1^
3. Zion, UT	PJ	37.2	− 113.0**	2000	374	95	^1^
4. Grand Canyon, AZ	PJ	34.7	− 112.4*	2120	367	123	^1^
5. Pinedale, AZ	PJ	34.3	− 110.3**	1970	427	183	^1^
6. Blue, AZ	P	33.6		1980	490	286	^1^
7. Los Pinos Mountains, NM_a_	PJ*	34.3	− 106.5	1911	363	–	^2^
7. Los Pinos Mountains, NM_b_	PJ*	34.3	− 106.5	1911	443	–	^2^
7. Los Pinos Mountains, NM_c_	PJ*	34.3	− 106.5	1911	200	–	^2^
8. Pajarito Plateau, NM	PJ*	35.8	− 106.3*	2150	470	–	^3^

“P” represents *Pinus edulis* and “J” represents either *Juniperus monosperma* (*NM sites) or *J. osteosperma,* which hybridize within Arizona and Utah. References: 1) Williams & Elheringer 2000; 2) Limousin et al*.* 2013; 3) Garcia-Forner et al. 2016; a = ambient, b = irrigation, and c = drought treatments from Limousin et al*.* 2013. Longitudes denoted with ‘**’ were estimated when actual longitude was not provided in the associated reference.

### Statistical analyses

To assess statistical significance in SCP differences between rehydration treatments for each species within our short-term rehydration experiment, we used species-specific linear models with overnight rehydration and initial rehydration treatment as fixed effects using an alpha critical value of 0.05 to determine statistical significance. We performed statistical analyses using the “car”^65^ and “emmeans”^66^ packages with R software^67^ for linear regression and Tukey’s HSD test for post‐hoc analysis. Assumptions of the linear model were verified using a Shapiro–wilk normality test and Breusch-Pagan homoscedasticity test using the base and “lmtest”^68^ package, respectively.

To assess the response of piñon pine trees to regular watering at the seasonal time scale, we used a linear model with weeks since the beginning of the experiment and time of measurement pre-dawn or midday as fixed effects to determine the point at which Ψ_pd_ and Ψ_md_ differed significantly for more than two consecutive weeks. The point in time when Ψ_pd_ and Ψ_md_ significantly differ was used to separate “Zone 1” (i.e., Ψ_pd_ and Ψ_md_ do not differ significantly) from “Zone 2” (i.e., Ψ_md_ remains significantly lower than Ψ_pd_). We then tested the significance of differences in Ψ_md_ between “Zone 1” and “Zone 2” acclimation periods using Welch’s Two Sample t-test.

Finally, to assess the response of piñon and juniper SCP to variations in MAP at the ecosystem scale, we used a linear model with species as fixed effects using an alpha critical value of 0.05 to determine statistical significance in R^61^. We used a model reduction approach, starting with a full interaction model (lm(P_g12_ ~ MAP * Species)), dropping interactions and/or parameters (i.e., Species) that were not significant, and reporting the final, reduced-order model (see SI Table 2). Finally, eta squared (η2) was calculated to estimate the effect size of each predictor variable and their interaction^69^ using the “lsr”^70^ package in R.

## Electronic supplementary material

Below is the link to the electronic supplementary material.

Supplementary Material 1

Supplementary Material 2

## Data Availability

All data generated or analysed during this study are included in this published article (and its Supplementary Information files).
