# Supplementary material for: Increased water availability at various timescales has different effects on stomatal closure point in isohydric piñon pine and anisohydric juniper
Source: Sci Rep. 2025 May 12;15:16476. doi: 10.1038/s41598-025-00582-6 (PMC12069681; doi:10.1038/s41598-025-00582-6)
Supplement: Supplementary file 1 — Supplementary Material 1 [file 41598_2025_582_MOESM1_ESM.docx]

**Dataset 1:** **SCP data used in our branch-level experiment.** We determined SCP by generating stomatal dehydration response curves for branchlets collected from excised branches of mature, field-grown piñon pine and juniper (*Juniperus monosperma* only) measured both immediately after sampling and after short-term rehydration (i.e., hourly to daily). SCPs were measured using the standard benchtop dehydration method that examines the relationship between G_s_ and Ψ_leaf_ to generate stomatal dehydration response curves^1,2^. The first two digits in the tree ID refer to the sampled trees that were also target trees in the ambient control treatment of the Survival and Mortality (SUMO) experiment in Los Alamos, NM^3^.

| **Tree ID** | **Species** | **Overnight Rehydration** | **Initial**  **Rehydration** | **Pg12_MPa** |
| --- | --- | --- | --- | --- |
| J2.1 | Juniper | No | No | -3.789 |
| J2.2 | Juniper | No | No | -2.814 |
| J2.3 | Juniper | Yes | Yes | -3.173 |
| J2.4 | Juniper | Yes | Yes | -3.292 |
| J2.5 | Juniper | Yes | Yes | -3.157 |
| J2.6 | Juniper | No | No | -2.692 |
| J2.7 | Juniper | No | Yes | -2.639 |
| J2.8 | Juniper | No | Yes | -4.095 |
| J2.9 | Juniper | No | Yes | -3.851 |
| J2.10 | Juniper | Yes | No | -3.684 |
| J2.11 | Juniper | Yes | No | -3.614 |
| J2.12 | Juniper | Yes | No | -3.221 |
| P1.1 | Piñon | No | No | -3.448 |
| P1.2 | Piñon | Yes | Yes | -5.539 |
| P1.3 | Piñon | Yes | Yes | -3.625 |
| P1.4 | Piñon | Yes | Yes | -4.418 |
| P1.5 | Piñon | No | No | -3.276 |
| P1.6 | Piñon | No | No | -3.559 |
| P1.7 | Piñon | No | Yes | -2.694 |
| P1.8 | Piñon | No | Yes | -3.085 |
| P1.9 | Piñon | No | Yes | -2.733 |
| P1.10 | Piñon | Yes | No | -3.641 |
| P1.11 | Piñon | Yes | No | -3.392 |
| P1.12 | Piñon | Yes | No | -4.414 |

**Dataset 2:** **SCP data used in our tree-level experiment.** We used midday (minimum) leaf water potential (Ψ_min_)4 as a proxy for SCP of mature, piñon pine trees transplanted into pots in a greenhouse and exposed to regular watering (see control treatment in Sevanto et al.^14^). Specifically, five mature, 2-2.5m tall piñon pine trees were transplanted in 86-liter pots and irrigated daily to field capacity with tap water. After acclimatizing for a three-month period, Ψ_leaf_ was measured on two branches per tree using pressure chamber (Model 1005, PMS Instrument Company, Albany, OR, USA)^58^, at pre-dawn (Ψ_pd_) and mid-day (Ψ_md_) weekly for approximately ten months, from 25 March 2010 to 2 Feb 2011^14^. Tree IDs refer to the control tree in the experiment of Sevanto et al. (2014)^5^.

| **Predawn (-bar)** | | | | | |
| --- | --- | --- | --- | --- | --- |
| **Date** | **Tree**  **ID1** | **Tree ID3** | **Tree ID6** | **Tree ID13** | **Tree ID15** |
| 1/26/2010 | 5.7 | 4.1 | 3.5 | 8 | 2.6 |
| 1/26/2010 | 6.5 | 11.5 | 6.4 | 10.3 | 8.4 |
| 2/11/2010 | 5 | 7 | 11.5 | 4.5 | 20 |
| 2/11/2010 | 7 | 10 | 26 | 8.5 | 20 |
| 3/18/2010 | 3.6 | 8.8 | 5.4 | 3.9 | 15.4 |
| 3/18/2010 | 5.5 | 10.9 | 14.6 | 6.6 | 15.9 |
| 3/30/2010 | 5.9 | 2.5 | 8.3 | 1.3 | 5.6 |
| 3/30/2010 | 6.4 | 3.4 | 11.6 | 3.8 | 11.2 |
| 4/5/2010 | 9.3 | 2.3 | 2.9 | 2.9 | 14.1 |
| 4/5/2010 | 9.5 | 6.8 | 3.1 | 3.1 | 15.2 |
| 4/14/2010 | 4.1 | 2 | 2.2 | 5.1 | 3.8 |
| 4/14/2010 | 4.5 | 4.2 | 6.9 | 6.2 | 10.5 |
| 4/21/2010 | 3.5 | 1.8 | 5.3 | 4.7 | 5.9 |
| 4/21/2010 | 4.3 | 5.3 | 9.6 | 12.2 | 8.4 |
| 4/29/2010 | 3.5 | 3.7 | 3.6 | 2.7 | 3 |
| 4/29/2010 | 6.8 | 11.9 | 10.8 | 7.3 | 5.4 |
| 5/5/2010 | 3.3 | 5.1 | 3.2 | 5 | 9.1 |
| 5/5/2010 | 6.7 | 5.6 | 8.7 | 8.2 | 11.4 |
| 5/12/2010 | 9.8 | 8.1 | 7.5 | 9.8 | 9 |
| 5/12/2010 | 13.5 | 9.1 | 8.5 | 10.3 | 9.6 |
| 5/19/2010 | 2.8 | 5.1 | 4.4 | 5.6 | 12.2 |
| 5/19/2010 | 3.5 | 6.7 | 8.9 | 10.2 | 14 |
| 5/26/2010 | 8.2 | 5.3 | 7.4 | 8.1 | 8.9 |
| 5/26/2010 | 9 | 9.1 | 11.8 | 8.6 | 9.6 |
| 6/2/2010 | 8.8 | 9.6 | 8.3 | 9.9 | 8.7 |
| 6/2/2010 | 9.9 | 13.7 | 9.7 | 10.1 | 9.6 |
| 6/9/2010 | 9.6 | 8.4 | 6.3 | 8.9 | 10.2 |
| 6/9/2010 | 9.8 | 9.1 | 8.7 | 9.2 | 10.6 |
| 6/16/2010 | 11 | 9 | 11 | 13.1 | 10.3 |
| 6/16/2010 | 16 | 10 | 13 | 16.8 | 11 |
| 6/23/2010 | 9.6 | 8.6 | 8.4 | 10 | 9.7 |
| 6/23/2010 | 10.1 | 9.7 | 8.6 | 11.8 | 10.1 |
| 6/30/2010 | 9.9 | 8.1 | 8.4 | 10 | 8.4 |
| 6/30/2010 | 10.2 | 10.3 | 8.9 | 10 | 11.3 |
| 7/7/2010 | 9 | 8.3 | 7.6 | 9.2 | 9.4 |
| 7/7/2010 | 9.4 | 8.5 | 8.2 | 9.6 | 10.5 |
| 7/14/2010 | 9.3 | 9 | 8.9 | 11.5 | 11.4 |
| 7/14/2010 | 9.5 | 9.3 | 8.9 | 12.6 | 14.8 |
| 7/21/2010 | 10.6 | 10.6 | 7.7 | 9.9 | 9.1 |
| 7/21/2010 | 12.1 | 12.9 | 8.2 | 10.3 | 14 |
| 7/28/2010 | 12.2 | 9.7 | 9.9 | 11 | 9.9 |
| 7/28/2010 | 13.1 | 11 | 17.4 | 11.8 | 18.4 |
| 8/4/2010 | 8.9 | 8.3 | 7.6 | 9.3 | 8.4 |
| 8/4/2010 | 10.3 | 8.3 | 7.8 | 14.9 | 8.8 |
| 8/11/2010 | 10.7 | 11.4 | 8.7 | 10.3 | 12.5 |
| 8/11/2010 | 11.5 | 12.9 | 10.4 | 11.6 | 13.1 |
| 8/18/2010 | 18.7 | 12.9 | 13.3 | 13.8 | 11.5 |
| 8/18/2010 | 19.5 | 13.8 | 14 | 14.2 | 13.3 |
| 8/25/2010 | 12.8 | 13.4 | 10.4 | 15 | 4.6 |
| 8/25/2010 | 13.6 | 17.6 | 10.9 | 16.2 | 10.9 |
| 9/1/2010 | 11.6 | 10.7 | 11.4 | 8.8 | 9.9 |
| 9/1/2010 | 12.3 | 11.4 | 15.8 | 14.5 | 11.3 |
| 9/8/2010 | 12.6 | 10.3 | 11.2 | 9.9 | 8.9 |
| 9/8/2010 | 14.2 | 11.1 | 13.7 | 10.2 | 9.1 |
| 9/15/2010 | 9.6 | 10.7 | 8.8 | 10.1 | 8.4 |
| 9/15/2010 | 14.2 | 11.2 | 9.3 | 10.2 | 8.6 |
| 9/22/2010 | 10.8 | 8.3 | 8.7 | 8.4 | 7.3 |
| 9/22/2010 | 15.6 | 8.4 | 9.1 | 8.6 | 8.1 |
| 9/29/2010 | 11.3 | 9.7 | 11.8 | 12.9 | 15.4 |
| 9/29/2010 | 18.9 | 10.3 | 14.7 | 16.7 | 15.4 |
| 10/5/2010 | 10.6 | 7.8 | 11.9 | 8.5 | 7.4 |
| 10/5/2010 | 11.8 | 9.4 | 11.8 | 24.8 | 7.4 |
| 10/14/2010 | 14.8 | 9.8 | 9.6 | 10.9 | 9.9 |
| 10/14/2010 | 16.4 | 11.9 | 10.2 | 11.6 | 11.3 |
| 10/20/2010 | 13.2 | 11.4 | 14.1 | 12.5 | 8.6 |
| 10/20/2010 | 13.9 | 12 | 16.7 | 12.5 | 9.5 |
| 10/27/2010 | 9.2 | 6.5 | 11.8 | 7.7 | 7.5 |
| 10/27/2010 | 9.9 | 10.6 | 12.6 | 9.6 | 8 |
| 11/2/2010 | 13 | 10.5 | 7.8 | 6.7 | 9.8 |
| 11/2/2010 | 11.6 | 13.8 | 10.8 | 14.6 | 10.1 |
| 11/10/2010 | 8.7 | 10.6 | 12.4 | 10.4 | 8 |
| 11/10/2010 | 10 | 10.9 | 18.8 | 33.8 | 8 |
| 11/17/2010 | 11.6 | 8.9 | 6.3 | 6.5 | 7.5 |
| 11/17/2010 | 11.6 | 9.5 | 6.7 | 9.8 | 9.5 |
| 11/24/2010 | 8 | 7.7 | 6.6 | 9.8 | 7 |
| 11/24/2010 | 9.6 | 8.2 | 7.7 | 9.7 | 7 |
| 12/1/2010 | 8.8 | 7.8 | 6.3 | 6.7 | 6.6 |
| 12/1/2010 | 10.1 | 8.4 | 6.6 | 6.9 | 6.8 |
| 12/8/2010 | 8.8 | 10.1 | 6.8 | 8.6 | 11.2 |
| 12/8/2010 | 9.8 | 10.5 | 7.5 | 9.8 | 11.4 |
| 12/15/2010 | 8.1 | 7.5 | 5.8 | 5.7 | 6.7 |
| 12/15/2010 | 8.2 | 8.3 | 6.3 | 6.1 | 6.9 |
| 12/22/2010 | 8.5 | 7.2 | 7.1 | 6.9 | 7.4 |
| 12/22/2010 | 8.9 | 7.7 | 7.3 | 7.8 | 8.5 |
| 12/29/2010 | NA | NA | NA | NA | NA |
| 12/29/2010 | NA | NA | NA | NA | NA |
| 1/5/2011 | 9.8 | 6.6 | 6.6 | 7.9 | 7.6 |
| 1/5/2011 | 10.2 | 8.6 | 8.9 | 9.6 | 9 |
| 1/12/2011 | 8.2 | 6.4 | 6.2 | 6.5 | 7.6 |
| 1/12/2011 | 8.7 | 6.7 | 8.8 | 7.4 | 8.2 |
| 1/19/2011 | 9.4 | 6.8 | 6.1 | 8 | 5.9 |
| 1/19/2011 | 9.6 | 10.6 | 6.7 | 8.4 | 6.8 |
| 1/26/2011 | 7.5 | 6.9 | 6.4 | 7.6 | 7.8 |
| 1/26/2011 | 8.2 | 7.4 | 6.7 | 8.7 | 8.6 |
| 2/2/2011 | 8.8 | 6.3 | 6.5 | 6.9 | 6.3 |
| 2/2/2011 | 10.7 | 6.7 | 7.2 | 7.2 | 7 |

| **Midday (-bar)** | | | | | |
| --- | --- | --- | --- | --- | --- |
| **Date** | **Tree ID1** | **Tree ID3** | **Tree ID6** | **Tree ID13** | **Tree ID15** |
| 1/26/2010 | NA | NA | NA | NA | NA |
| 1/26/2010 | NA | NA | NA | NA | NA |
| 2/11/2010 | NA | NA | NA | NA | NA |
| 2/11/2010 | NA | NA | NA | NA | NA |
| 3/18/2010 | 22 | 22 | 21.5 | 19 | 8.5 |
| 3/18/2010 | 25 | 25.7 | 23.5 | 21.5 | 16.8 |
| 3/30/2010 | 3.6 | 5.7 | 7.4 | 7.8 | 4.8 |
| 3/30/2010 | 5 | 6.6 | 14.9 | 24.6 | 7.6 |
| 4/5/2010 | 4.2 | 8.3 | 8.5 | 10.9 | 2 |
| 4/5/2010 | 6.8 | 13.9 | 25.5 | 14.2 | 11.2 |
| 4/14/2010 | 2.7 | 1.8 | 8.4 | 6.4 | 7.2 |
| 4/14/2010 | 7.9 | 5.9 | 13.3 | 11.7 | 16 |
| 4/21/2010 | 8.5 | 1.5 | 4.3 | 28.9 | 11.7 |
| 4/21/2010 | 9.7 | 13.6 | 21.5 | 28.4 | 13.5 |
| 4/29/2010 | 5.8 | 2.6 | 6 | 3.4 | 11.8 |
| 4/29/2010 | 6.4 | 4.1 | 14.8 | 5.2 | 19.2 |
| 5/5/2010 | 3.8 | 5.2 | 28.8 | 7.6 | 10 |
| 5/5/2010 | 18.7 | 6.6 | 12.6 | 9.8 | 14.3 |
| 5/12/2010 | 27.1 | 20.2 | 23.6 | 21.3 | 23.5 |
| 5/12/2010 | 28.4 | 22.2 | 21.1 | 22.6 | 25.9 |
| 5/19/2010 | 7.8 | 7.6 | 7.7 | 11.7 | 5.5 |
| 5/19/2010 | 13 | 10.2 | 18.2 | 12.5 | 7.4 |
| 5/26/2010 | 17.9 | 7.6 | 6.4 | 18 | 21.2 |
| 5/26/2010 | 19.5 | 10.8 | 34.9 | 21.9 | 23.6 |
| 6/2/2010 | 25.5 | 24.8 | 25.3 | 21.6 | 24.3 |
| 6/2/2010 | 25.5 | 26.3 | 26.9 | 23.4 | 25.5 |
| 6/9/2010 | 26.7 | 17.3 | 22.5 | 20 | 27.1 |
| 6/9/2010 | 25.8 | 23.1 | 27.1 | 23.9 | 28.3 |
| 6/16/2010 | 9.9 | 19.4 | 35.2 | 22.1 | 23.7 |
| 6/16/2010 | 26.5 | 29.9 | 47.1 | 28.3 | 25.2 |
| 6/23/2010 | 24 | 14.5 | 31.1 | 22.2 | 22.7 |
| 6/23/2010 | 29.1 | 25.5 | 32.4 | 23.6 | 24.1 |
| 6/30/2010 | 22.3 | 24.4 | 24.5 | 20.8 | 28.7 |
| 6/30/2010 | 23.6 | 38.6 | 28.6 | 21.9 | 31.5 |
| 7/7/2010 | 27.3 | 26.8 | 30.4 | 31.2 | 30.3 |
| 7/7/2010 | 30.3 | 30.7 | 30.4 | 31.8 | 37.6 |
| 7/14/2010 | 17.5 | 25.7 | 18.7 | 22.6 | 22.8 |
| 7/14/2010 | 18 | 26.8 | 23.8 | 23.5 | 27 |
| 7/21/2010 | 19 | 26.8 | 20.5 | 21.5 | 20.3 |
| 7/21/2010 | 22 | 19.2 | 23.5 | 22.5 | 20.9 |
| 7/28/2010 | 29.4 | 26.4 | 24.7 | 17.9 | 25.7 |
| 7/28/2010 | 37 | 28.7 | 25.6 | 28.7 | 26.2 |
| 8/4/2010 | 26.5 | 22.3 | 19.8 | 25.5 | 21.2 |
| 8/4/2010 | 30 | 29.4 | 33.6 | 26.5 | 26 |
| 8/11/2010 | 27.6 | 31.8 | 36 | 25.8 | 22 |
| 8/11/2010 | 31 | 32.5 | 33 | 30.6 | 25.8 |
| 8/18/2010 | 26.4 | 25.6 | 26.9 | 25.2 | 25.1 |
| 8/18/2010 | 28.2 | 27.3 | 27.9 | 29.3 | 26 |
| 8/25/2010 | 26.8 | 28.9 | 25 | 24.7 | 24.7 |
| 8/25/2010 | 33.1 | 39.1 | 39.8 | 26.5 | 26.8 |
| 9/1/2010 | 24.3 | 20.6 | 31.8 | 21.3 | 22.3 |
| 9/1/2010 | 26.4 | 27.7 | 37.9 | 27.9 | 24.5 |
| 9/8/2010 | 20.3 | 21.5 | 27.1 | 19.8 | 20.4 |
| 9/8/2010 | 21.2 | 23 | 23.7 | 24.9 | 21.8 |
| 9/15/2010 | 28 | 27 | 30 | 29.7 | 23.5 |
| 9/15/2010 | 28 | 27 | 30 | 31.6 | 26.3 |
| 9/22/2010 | 19 | 23.6 | 17 | 23.6 | 24 |
| 9/22/2010 | 21.4 | 38.8 | 24.9 | 24.1 | 25.2 |
| 9/29/2010 | 19.9 | 20.8 | 20.4 | 16.8 | 18.3 |
| 9/29/2010 | 23.6 | 22.9 | 25 | 19.1 | 18.3 |
| 10/5/2010 | 21.6 | 18.4 | 25.8 | 22.1 | 18.3 |
| 10/5/2010 | 24.6 | 21.6 | 27.2 | 26.4 | 23.2 |
| 10/14/2010 | 19.6 | 32.6 | 25.8 | 22.2 | 25.5 |
| 10/14/2010 | 34.9 | 32.6 | 27.4 | 22.2 | 21 |
| 10/20/2010 | 10 | 21 | 26 | 23 | 21.2 |
| 10/20/2010 | 11 | 22 | 27 | 29.5 | 23 |
| 10/27/2010 | 26.8 | 27.5 | 22.8 | 17.9 | 18.6 |
| 10/27/2010 | 28 | 28.2 | 26.5 | 23.9 | 20.6 |
| 11/2/2010 | 19.6 | 26.3 | 22.2 | 17.8 | 16.8 |
| 11/2/2010 | 20.8 | 27.5 | 23.1 | 22.1 | 18.9 |
| 11/10/2010 | 21.9 | 24.7 | 23.5 | 22.5 | 23.9 |
| 11/10/2010 | 28.4 | 26.6 | 25.4 | 27 | 25.2 |
| 11/17/2010 | 20.6 | 22.4 | 28.5 | 18.5 | 22.8 |
| 11/17/2010 | 24.9 | 28.5 | 30.4 | 20.2 | 27.9 |
| 11/24/2010 | 21 | 23.5 | 25 | 15.6 | 20.5 |
| 11/24/2010 | 25 | 23.5 | 27 | 19.2 | 22.8 |
| 12/1/2010 | 18.1 | 20.9 | 19.9 | 18.8 | 20.5 |
| 12/1/2010 | 21.2 | 22.1 | 20.5 | 19.2 | 21.1 |
| 12/8/2010 | 20.2 | 24.5 | 22.4 | 20.2 | 20.6 |
| 12/8/2010 | 25.1 | 26.9 | 23.7 | 22.1 | 21.2 |
| 12/15/2010 | 18.2 | 16.8 | 20.1 | 14.9 | 16.9 |
| 12/15/2010 | 21.1 | 19.4 | 21.3 | 20.8 | 17.2 |
| 12/22/2010 | 19.2 | 14.7 | 18.5 | 15.9 | 15.1 |
| 12/22/2010 | 19.6 | 16.9 | 20.2 | 16.3 | 15.4 |
| 12/29/2010 | NA | NA | NA | NA | NA |
| 12/29/2010 | NA | NA | NA | NA | NA |
| 1/5/2011 | 27.3 | 28.6 | 22.4 | 33.4 | 19.9 |
| 1/5/2011 | 28.9 | 31.9 | 25.8 | 34.2 | 24.6 |
| 1/12/2011 | 22.6 | 19.9 | 21.4 | 18.8 | 19.3 |
| 1/12/2011 | 22.9 | 19.9 | 23.7 | 22 | 20.7 |
| 1/19/2011 | 22.4 | 22.3 | 28.7 | 22.8 | 19.6 |
| 1/19/2011 | 23.5 | 23.3 | 29.9 | 22.8 | 24.4 |
| 1/26/2011 | 21.8 | 24.3 | 19.7 | 20.8 | 16.8 |
| 1/26/2011 | 22.8 | 25.1 | 22.5 | 24.3 | 22.6 |
| 2/2/2011 | 22 | 17.8 | 18.7 | 25.4 | 21.1 |
| 2/2/2011 | 27.6 | 18.9 | 20.3 | 23.2 | 22.8 |

**References:**

1. Hinckley, T. M., Duhme, F., Hinckley, A. R. & Richter, H. Water relations of drought hardy shrubs: osmotic potential and stomatal reactivity. *Plant Cell Environ.* **3**, 131–140 (1980).

2. Skelton, R. P., West, A. G. & Dawson, T. E. Predicting plant vulnerability to drought in biodiverse regions using functional traits. *Proc. Natl. Acad. Sci.* **112**, 5744–5749 (2015).

3. Garcia-Forner, N. *et al.* Responses of two semiarid conifer tree species to reduced precipitation and warming reveal new perspectives for stomatal regulation. *Plant Cell Environ.* **39**, 38–49 (2016).

4. Knipfer, T. *et al.* Predicting Stomatal Closure and Turgor Loss in Woody Plants Using Predawn and Midday Water Potential. *Plant Physiol.* **184**, 881–894 (2020).

5. Sevanto, S., Mcdowell, N. G., Dickman, L. T., Pangle, R. & Pockman, W. T. How do trees die? A test of the hydraulic failure and carbon starvation hypotheses. *Plant Cell Environ.* **37**, 153–161 (2014).
