# Supplementary material for: Increased water availability at various timescales has different effects on stomatal closure point in isohydric piñon pine and anisohydric juniper
Source: Sci Rep. 2025 May 12;15:16476. doi: 10.1038/s41598-025-00582-6 (PMC12069681; doi:10.1038/s41598-025-00582-6)
Supplement: Supplementary file 2 — Supplementary Material 2 [file 41598_2025_582_MOESM2_ESM.docx]

**SI Table 1. Summary of linear interaction model coefficients for SCP as a function of summer precipitation.**

*Note: lm(P_g12_ ~ Precip_Jul.Sep * Species), adjusted R^2^ = 0.3837, p = 0.126.*

|  | **Estimate** | **Std. Error** | **pr(>\|t\|)** | **numDF** |
| --- | --- | --- | --- | --- |
| **Intercept** | -3.625094 | 0 | 0.00252 | 6 |
| **Precip_Jul.Sep** | 0.006545 | 0 | 0.32820 | 6 |
| **SpeciesPine** | 0.795421 | 0 | 0.41566 | 6 |
| **Precip_Jul.Sep:SpeciesPine** | -0.001867 | 0 | 0.79656 | 6 |

**SI Table 2. Summary of linear interaction model coefficients for SCP as a function of mean annual precipitation.** Note: reduced order model is presented in the main document as interaction is not significant.

*Note: lm(P_g12_ ~ MAP * Species), adjusted R^2^ = 0.6094, p = 0.0009491.*

|  | **Estimate** | **Std. Error** | **pr(>\|t\|)** | **numDF** |
| --- | --- | --- | --- | --- |
| **Intercept** | -5.394718 | 0 | 2.83e-05 | 14 |
| **Mean Ann. Precip.** | 0.005559 | 0 | 0.0278 | 14 |
| **SpeciesPine** | 2.692657 | 1 | 0.0453 | 14 |
| **MAP:SpeciesPine** | -0.003997 | 0 | 0.219 | 14 |
